# Utilization of abortion services from an unsafe provider and associated factors among women with history of induced abortion in Ghana

**DOI:** 10.1186/s12884-022-05034-x

**Published:** 2022-09-13

**Authors:** Desmond Klu, Isaac Yeboah, Esinam Afi Kayi, Joshua Okyere, Mary Naana Essiaw

**Affiliations:** 1 Institute of Health Research, University of Health and Allied Sciences, PMB 31, Ho, Volta Region Ghana; 2 Institute of Work, Employment and Society, University of Professional Studies, P.O. Box LG 149, Legon Accra, Ghana; 3 Department of Adult Education and Human Resource Studies, School of Continuing and Distance Education, University of Ghana, P.O. Box LG 25, Accra, Legon Ghana; 4 Department of Population and Health, University of Cape Coast, PMB 40, Cape Coast, Ghana

**Keywords:** Abortion, Unsafe provider, Factors, Utilization, Induced abortion, Ghana

## Abstract

**Background:**

In sub-Saharan Africa (SSA), numerous studies have examined women’s choice of abortion methods and services using hospital-based data, community-based surveys and nationally representative data. Little research focuses on the factors influencing a woman’s choice of abortion provider. This study sought to identify factors that are associated with why a woman seeks abortion care services from an unsafe provider in Ghana.

**Methods:**

We used nationally representative data of women from the 2017 Ghana Maternal Health Survey (GMHS). Data analysis was restricted to women aged 15–49 with a recent history of induced abortion. Analyses focused on a weighted sample of 1,880. Descriptive analysis and the chi-square test were used to examine the proportion of women utilizing abortion services from unsafe providers. Factors hypothesized to affect the utilization of abortion services from unsafe providers were examined using both bivariable and multivariable logistic regression analyses.

**Results:**

The proportion of survey respondents who reported that they utilize abortion service from unsafe providers were 57.5%. After adjusting for confounders, those who have knowledge of abortion legality [aOR: 0.381 (0.271–0.541)] and those who have attained secondary or higher education [aOR: 0.613 (0.411–0.914)] were less likely to use abortion services from unsafe providers. On the other hand, women belonging to the Ewe ethnic group [aOR: 0.696 (0.508–0.953)], those residing in the middle belt zone [aOR: 1.743 (1.113–2.728)], younger women aged 15–29 years [aOR: 2.037 (1.234–3.362)] were more likely to use abortion services from unsafe abortion providers.

**Conclusions:**

This research suggests that increasing the knowledge of women on the legal status of abortion through public education and encouraging more women to pursue secondary or higher education can contribute to reducing the use of abortion services from unsafe providers. These interventions should be targeted among younger women and those who reside in the middle belt zones of Ghana.

## Background

The availability and access to well-trained, highly skilled and medically certified health care providers is crucial in reducing the public health burden of unsafe abortion [1, 2]. According to Henkel and Shaw [3], abortion is safe when it is performed by an “appropriately trained health care provider with methods recommended by World Health Organization (WHO)”; it is less safe when it is provided by “trained providers using non recommended (e.g., sharp curettage) methods or using a safe method (e.g., misoprostol) but without adequate information or support from a trained individual”; and it is least safe when it is provided by “untrained people using dangerous, invasive methods”. Both the less safe and least safe are compositely referred to as unsafe abortion. Unsafe abortion contributes to approximately 8 percent of maternal-related deaths and is hence a major cause of morbidity in women [4].

Studies have shown that the choice of the services of abortion provider influences comprehensive abortion and post abortion care services. A study by Maxwell et al. [5] asserted that women who received abortion services from safe providers (midwives and physicians) are more likely to receive a long-acting and permanent contraceptive method than a short-acting contraceptive method. Using safe abortion providers provides an opportunity to plan pregnancy to improve the health status of women [1]. In contrast, abortion services provided by unsafe providers are associated with economic, health and social consequences, including an increased risk of infant mortality, abortion-related mortalities and sometimes unresolved health complications [2, 4].

Globally, the use of unsafe and unapproved abortion providers is more prevalent in settings where access to legal abortion services is highly controlled [6]. Ghana is one of the few sub-Saharan African countries with a liberal abortion law that allows abortion in cases of rape, incest, foetal abnormalities or when the life of the mother or unborn child is at risk [7–9]. Despite the liberalization of the law, reliance on unsafe abortion providers increased from 43 percent in 2007 to 59 percent in 2017 [10]. To reduce the incidence of maternal morbidity and mortality as a result of unsafe abortion, the Ghana Health Service implemented the R3M (Reducing Maternal Mortality and Morbidity) programme [8, 10] in conjunction with a consortium of five organizations (EngenderHealth, Ipas, Marie Stopes International, Population Council, and the Willows Foundation). Among others, the programme aimed at improving access to family planning services and comprehensive abortion care services [8]. These efforts, however, have minimally yielded a significant impact on reducing the proportion of women who utilize the services of unsafe providers in Ghana [11].

A clear comprehension of the factors that influence the preference for unsafe abortion service providers is important, especially in the context where access to abortion services is limited or where myths and misconceptions surrounding abortion are viewed as negative and where negative attitudes of health care providers toward abortion care services are rife. Moreover, there is high recognition of the extent to which the choice of abortion providers implicates the sexual and reproductive lives of women and the health care system [12].

Empirically, a large body of literature documents the determinants of the choice of safe methods [8, 9, 13] and unsafe abortion methods [14, 15]. Other studies also examine the association between women’s knowledge of abortion law and the practice of unsafe abortion services [16, 17]. Furthermore, while some studies use hospital-based data to examine this phenomenon [18, 19] and community-based surveys [18, 20], others use nationally representative surveys [15, 21]. These previous studies did not fully explore and examine how demographic and socioeconomic factors influence women’s choice of unsafe abortion providers. Again, to the best of our knowledge, no study in Ghana has investigated the factors that influence a woman’s choice of abortion provider using nationally representative data. Understanding the factors that influence utilization of abortion care services from unsafe providers can help design interventions to reach women who are at higher risk of infant mortality and abortion related complications.

The present study seeks to identify and investigate the demographic and socioeconomic factors that influence women’s choice of unsafe abortion provider in Ghana using the 2017 Ghana Maternal Health Survey (GMHS). The study will also assess the utilization of abortion care services from unsafe abortion providers among women with a history of induced abortion in the past five years preceding the survey. The finding from the study is fundamental to achieving the Sustainable Development Goals (SDGs) on health wellbeing (SDG3) which recognizes access to sexual and reproductive health information and services.

## Methods and Materials

The study utilized secondary data from the 2017 (GMHS). The 2017 GMHSs were undertaken by the Ghana Statistical Service (GSS) with technical support from Inner City Fund (ICF) Macro International through the DHS Program, funded by the United States Agency for International Development (USAID), Government of Ghana, the European Union (EU) and the United Nations Population Fund (UNFPA). The study received ethical approval from the ICF Macro Institutional Review Board, Maryland, USA. The sampling frame adopted was from the 2010 Ghana Population and Housing Censuses (PHCs). The GMHS used a multistage stratified cluster sampling method to select the eligible enumeration areas and households. Further details of the survey methodological and sampling procedures and questionnaires used can be accessed in the final report [22–25]. Women of reproductive age 15–49 years who were permanently resident in the selected household a night before the survey were eligible to be interviewed. For the purposes of this study, the women’s data file for the 2017 GHMS was used and the analysis is, limited to a subpopulation of women who terminated a pregnancy within five years preceding the survey. A total (weighted) sample of 1,880 women whose activities related to their most recent induced abortion were selected.

## Definition of study variables

### Outcome variable

The outcome variable is the choice of abortion provider. “Who did you see to get this first step (pregnancy termination) done? The responses were doctor, nurse/midwife, community health officer/nurse, pharmacist/chemical seller, traditional birth attendant, community health/volunteer, relative/friend, traditional practitioner, no one and other (specify). It was categorized in a binary form as “0” for “safe” and “1” for “unsafe” from the variable ‘type of abortion provider’. The ‘safe’ abortion providers comprised medical doctors, nurses or midwives. ‘Unsafe’ abortion providers consisted of nonmedically certified providers such as Auxiliary Midwife, Pharmacist/Chemical seller, Traditional Birth Attendant, Community Health Worker, Relative/Friend and Traditional Practitioner. Any woman who relied on the services of any of these providers to terminate the pregnancy was classified to have used either safe or unsafe abortion providers. This classification was guided by a similar classification used by Boah et al. [26] in their study on abortion in Ghana.

### Explanatory variables

Nine explanatory variables were considered in this study. These explanatory variables in the study included knowledge on legality of abortion, age, religious affiliation, marital status, educational level, ecological zones of residence, place of residence (rural or urban), ethnicity and parity (number of surviving children). Regarding age, those under age (15–17 years) were merged with adult women (20–29 years) for two reasons. First, the sample size of adolescent girls was too small. Second, the adolescent girls (15–19 years), young adult (20–24 years) and older adult (25–29 years) constitute young women. Hence, we wanted to find out how age of young women is associated with unsafe abortion providers.

The variable ecological zone was categorized from the 10 regions in Ghana into coastal zones (Western, Central, Volta and Greater Accra Regions), middle belt zones (Eastern, Ashanti, and Brong Ahafo Regions), and northern zones (Upper East, Upper West and Northern Regions).

## Data analysis

We used descriptive statistics, including frequency and percentages to explore the categorical variables of respondents’ characteristics. A chi-square test was used to identify the distribution of abortion providers across categories of explanatory variables. Binary logistic regression analysis was performed in two models: the first model (model 1) was a bivariate analysis of the explanatory variable on the use of unsafe abortion provider. Model 2 adjusted for the effects of the other explanatory variables to ascertain the association between these independent variables and outcome variable, choice for unsafe abortion providers. Binary logistic regression was employed because our dependent variable (unsafe abortion provider) was measured as a binary factor. We presented the regression analysis results as crude odds ratios (cORs) and adjusted odds ratio (aORs), with their corresponding 95% confidence intervals (CIs) signifying the precision and significance of the reported OR. All statistical analyses were performed using STATA version 15.

## Results

### Descriptive results

**Fig. 1:**
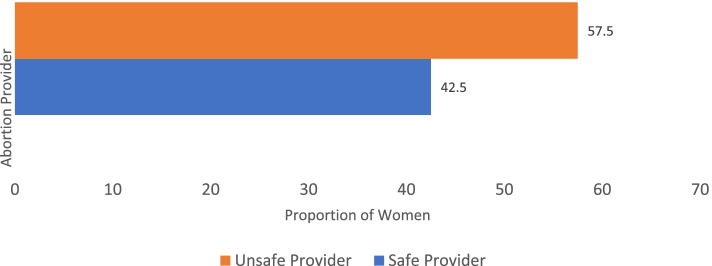
Proportion of women using the services of abortion providers

Figure 1 presents the proportion of women who used abortion care services from abortion providers. The figure indicates that the proportion of women who utilize abortion care services from unsafe providers was 57.5%.

Table 1 shows the proportion of women that utilize abortion care services from unsafe provider by explanatory variables. Respondents using unsafe abortion providers were significantly higher among those in the younger ages of 15–29 years [60.5%], those with no knowledge of abortion legality [60.0%], those affiliated with the Ga/Dangme ethnic groups [62.0%], those not married [61.6%], those with parity zero [60.7%] and those residing in the middle belt zone [62.4%]. The significant explanatory variables were significant at 0.001, except the parity and ecological zone of residence, with *p*-values < 0.05.

**Table 1:** Weighted proportion of women who utilize abortion service from abortion providers in different socio-demographic groups

Variables	Choice of abortion provider		
**Women’s knowledge on abortion legality**	Safe provider (%)	Unsafe provider (%)	*p*-value
Yes	63.7	36.3	< 0.001
No	40.0	60.0	
**Age**			
15–29	39.5	60.5	< 0.001
30–39	47.3	52.7	
40–49	53.8	46.2	
**Religious affiliation**			
Catholics	50.3	49.7	0.222
Protestants	40.6	59.4	
Moslem	43.8	56.3	
Pentecostal/Charismatic	42.2	57.8	
No religion	36.2	63.8	
**Educational level**			
No education	38.9	61.1	0.081
Primary	37.6	62.4	
Secondary +	43.9	56.1	
**Ethnicity**			
Akan	40.5	59.5	< 0.001
Ga/Dangme	38.0	62.0	
Ewe	52.8	47.2	
Mole-dagbani	40.9	59.1	
**Marital status**			
Married	50.8	49.2	< 0.001
Cohabiting	41.6	58.4	
Not married	38.4	61.6	
**Parity**			
0	39.3	60.7	0.001
1–2	40.5	59.5	
3 +	49.5	50.5	
**Ecological zone**			
Coastal zone	46.0	54.0	0.001
Middle belt	37.6	62.4	
Northern zone	49.1	50.9	
**Place of residence**			
Urban	43.8	56.2	0.111
Rural	40.0	60.0	

Source: Computed from Ghana maternal health surveys (GMHS) 2017

### Associations between explanatory variables and use of unsafe abortion providers among women who terminated pregnancy five years preceding the survey

Two models were fitted to examine the association between selected explanatory variables and use of abortion services from an unsafe provider, with the results presented in Table 2. Model 1 was a crude model that was unadjusted and model 2 adjusted for the confounders. In model 2, a statistically significant effect of use of unsafe abortion providers was found in some selected explanatory variables. From Table 2, younger women aged 15–29 years [aOR: 2.037 (1.234–3.362)], and those residing in the middle belt zone [aOR: 1.743 (1.113–2.728)] were more likely to use the services of unsafe abortion providers compared with older women aged 40–49 years and those residing in the northern belt zone respectively. The likelihood of using the services of unsafe abortion providers was significantly lower among those with knowledge of abortion legality [aOR: 0.381 (0.271–0.541)], those who had attained secondary or higher education [aOR: 0.613 (0.411–0.914)] and those belonging to the Ewe ethnic group [aOR: 0.696 (0.508–0.953)] compared with those with no knowledge on abortion legality, those with no formal education and those belonging to the Akan ethnic group respectively.

**Table 2:** Bivariable (unadjusted) and multivariable (adjusted) logistic regression analysis for unsafe abortion provider and its related factors

	**Model 1**		**Model 2**	
**Variables**	**Unadjusted model cOR (95% CI)**	***p***-value	**Adjusted model aOR (95% CI)**	***p***-value
**Knowledge on abortion legality** (ref = No)				
Yes	0.359(0.257–0.501)	< 0.001	0.383(0.271–0.541)	< 0.001
**Age of respondent** (ref = 40–49)				
15–29	2.153(1.410–3.289)	< 0.001	2.037(1.234–3.362)	0.005
30–39	1.434(0.913–2.254)	0.118	1.496(0.932–2.404)	0.095
**Place of residence** (ref = Urban)				
Rural	1.104(0.886–1.376)	0.377	1.058(0.835–1.340)	0.640
**Education** (ref = No education)				
Primary	1.148(0.754–1.747)	0.521	0.821(0.520–1.297)	0.398
Secondary +	0.894(0.637–1.255)	0.518	0.613(0.411–0.914)	0.016
**Marital status** (ref = Not married)				
Currently married	1.521(1.155–2.003)	0.003	1.168(0.864–1.579)	0.312
Currently cohabiting	1.651(1.264–2.157)	< 0.001	1.297(0.947–1.777)	0.104
**Parity** (ref = No children)				
1–2 children	0.953(0.743–1.222)	0.704	0.947(0.709–1.262)	0.708
3 or more	0.652(0.493–0.862)	0.003	0.810(0.548–1.197)	0.290
**Ethnicity** (ref = Akan)				
Ga/Dangme	1.120(0.732–1.716)	0.601	1.242(0.790–1.953)	0.348
Ewe	0.660(0.492–0.885)	0.006	0.696(0.508–0.953)	0.024
Mole-dagbani	0.793(0.608–1.034)	0.087	0.993(0.664–1.484)	0.971
**Religion** (ref = Catholic)				
Protestant	1.457(0.997–2.130)	0.052	1.363(0.905–2.051)	0.138
Moslems	1.143(0.727–1.799)	0.562	1.173(0.714–1.925)	0.529
Pentecostal/Charismatic	1.426(1.003–2.028)	0.048	1.305(0.892–1.908)	0.170
No religion	2.111(0.989–4.508)	0.054	2.206(0.993–4.902)	0.052
**Ecological Zone** (ref = Northern zone)				
Coastal zone	1.381(0.992–1.921)	0.055	1.438(0.913–2.265)	0.117
Middle-belt zone	1.782(1.276–2.496)	0.001	1.743(1.113–2.728)	0.015

Source: Computed from Ghana maternal health surveys (GMHS) 2017

Note: *cOR* is crude odds ratio; *aOR* is adjusted odds ratio; *CI* Confidence interval; Ref is reference category

## Discussion

The study assessed utilization of abortion care service from unsafe providers and associated factors among women with history of induced abortion within five years preceding the survey. This study used the 2017 GMHSs. The proportion of women who utilized abortion care services from unsafe providers was 57.5%. This suggests that the proportion of women utilizing abortion care services from unsafe abortion providers has shown a significant increase over the decade in Ghana compared with 37.1% of women who used abortion care services from unsafe providers in 2007. Women’s knowledge of abortion legality, education, age, ethnicity, and ecological zones were significantly associated with choosing abortion care services from unsafe provider. Specifically, women who indicated they had knowledge of the legality of abortion were less likely to use the services of unsafe abortion providers. This suggests the importance of abortion knowledge in guiding women to make informed and correct abortion decisions. Similar studies in Ghana also found that women who knew abortion legalities sought the services of safe abortion providers [27, 28]. The studies further indicate that women who have in-depth knowledge of the legality of abortion confidently go to recognized health facilities to seek abortion services without any fear of being stigmatized or maligned [28]. Knowledge on abortion legality would deepen women’s understanding of the reproductive health services and accessibility of services easier. The finding of this study suggests a need to incorporate sufficient state level mechanisms to allow women who qualify to undergo abortion. Moreover, women’s knowledge on abortion legality influence type of procedure and subsequently the type of provider to use.

The results from the study show that women with secondary or higher education are less likely to use the services of unsafe abortion providers than those with no formal education. Analogous studies have also associated safer abortion decisions with higher educational attainment of women [26, 28]. A plausible explanation for this finding could be that formal education serves as a conduit for empowering women and girls with accurate and comprehensive information about safe abortion [29]. As such, they become knowledgeable about the potential adverse effects of seeking unsafe abortion services; hence, they are able to make informed choices such as opting for the services of safe abortion providers. Moreover, through the empowerment that women and girls receive from formal education, they are likely to be unperturbed by societal stigma that often accompanies women’s safe abortion practices.

Consistent with previous studies from Nepal [30] and Pakistan [31], we found that younger women had a significantly higher likelihood of choosing unsafe abortion providers. A possible explanation for this finding could be the lack of or low level of knowledge about the adverse effects of unsafe abortion. Another likely explanation could be the age limit for obtaining legal abortion. In Ghana, minors (less than 18 years) cannot have a safe elective abortion without parental consent. This age restriction may influence younger women to seek alternatives to safe abortion services, thereby resorting to the services of unsafe abortion providers. It is possible that, for many young people, peer influences may contribute to influence decisions for the choice of abortion providers out of fears of being stigmatized or conforming to social pressures [32, 33].

Previous studies, such as that of Boah et al. [26], revealed that ecological zone was not significantly associated with induced abortions. However, our study found the contrast. Living in middle ecological zones was associated with an increased likelihood for women to seek the services of unsafe abortion providers compared to those in the northern ecological zone. It is worth noting that the middle zones are a migration destination for many women in the northern zone [34]. According to Baada, Baruah and Luginaah [35], the predominant economic activity in the northern zone is agriculture; as such, when they are out of season, women migrate to the middle and coastal zones to engage in mining activities and head porter business (popularly known as kayayei). These women who migrate to the middle zones are often exploited and raped, leading to pregnancy [36]. Due to their predicament, they may resort to choosing unsafe abortion providers since that option is usually devoid of many questioning from the providers and happens to be relatively inexpensive.

### Strengths and limitations of study

The data for this study were extracted from the GMHS, which is one of Ghana’s nationally representative datasets on women. Hence, our findings are generalizable to women of reproductive age in Ghana. Additionally, the GMHS has been used and validated by previous studies. As such, the reliability and validity of our results is undeniable. Nevertheless, there are some limitations that must be considered when interpreting and using our findings. First, the use of a secondary dataset that applied a cross-sectional design limits our analytical capacity. We are limited to only inferring the association between knowledge on abortion legality and the choice of abortion provider. We are unable to make any causal inferences. Additionally, we were limited to only data in the dataset. For that reason, the influence of cultural norms and expectations on the choice of abortion provider could not be assessed. The choice of abortion provider was self-reported; hence, there is the likelihood of recall bias.

## Conclusion

More than half of the women in this study had patronized the services of unsafe abortion providers. Knowledge of abortion legality, younger women, secondary or higher education, belonging to the Ewe ethnic group and living in the middle ecological zone were predictors of unsafe abortion provider. Protective factors of unsafe abortion providers were knowledge of abortion legality and secondary or higher education while risk factor for choosing unsafe abortion providers are being younger women, belonging to the Ewe ethnic group and residing in middle ecological zone.

### Policy implications

The findings of this study hold some policy implications for Ghana and other sub-Saharan Africa with similar characteristics. Generally, women with secondary or higher education were less likely to utilize the services abortion services from unsafe abortion providers, which shows the need to target women without formal education. Health improvement strategies with a focus on increasing knowledge of abortion legality across the country should be designed and implemented. As a short-term goal, training of all staff at the sexual and reproductive health unit at health facilities is highly recommended. With increased public education on legality of abortion and increased educational attainment among women, the use of unsafe abortion providers may decrease, which will ensure quality sexual and reproductive health and save women’s lives. Policies and interventions by the Ghana Health Service to increase safe abortion services and decrease the services of unsafe abortion providers should prioritize younger women (15–29 years) and those residing in the middle ecological zones.

## Data Availability

The datasets generated and/or analyzed during the current study are available in the https://www.dhsprogram.com/data/dataset/Ghana_Special_2017.cfm?flag=0 repository.

## Abbreviations

aOR: Adjusted odds ratio
CI: Confidence intervals
cOR: Crude odds ratio
EU: European union
GMHS: Ghana maternal health survey
ICF: Inner city fund
OR: Odds ratio
PHC: Population and housing censuses
R3M: Reducing maternal mortality and morbidity
SDG: Sustainable development goal
SSA: Sub-Saharan Africa
USAID: United States agency for international development
UNFPA: United Nations population fund
WHO: World health organization
